# Kidney involvement and histological findings in two pediatric COVID-19 patients

**DOI:** 10.1007/s00467-021-05212-7

**Published:** 2021-08-18

**Authors:** Jessica Serafinelli, Antonio Mastrangelo, William Morello, Valeria Fanny Cerioni, Adib Salim, Manuela Nebuloni, Giovanni Montini

**Affiliations:** 1 Pediatric Nephrology, Dialysis and Transplant Unit, Fondazione IRCCS Ca’ Granda - Ospedale Maggiore Policlinico Di Milano, Via Commenda 9, 20122 Milan, Italy; 2 ATS Monza E Brianza, Lombardy, Italy; 3 ATS Bergamo, Lombardy, Italy; 4 Pathology Unit, ASST Fatebenfretalli-Sacco, Department of Biological and Clinical Sciences, University of Milan, Milan, Italy; 5 Department of Clinical Sciences and Community Health, University of Milan, Milan, Italy

**Keywords:** SARS-CoV-2, Kidney biopsy, Children, Tubular damage, Glomerulonephritis

## Abstract

**Background:**

Histological findings of kidney involvement have been rarely reported in pediatric patients with SARS-CoV-2 infection. Here, we describe clinical, laboratory, and histological findings of two pediatric cases with almost exclusive kidney involvement by SARS-CoV-2.

**Results:**

A 10-year-old girl with IgA vasculitis nephritis underwent kidney biopsy, showing diffuse and segmental mesangial-proliferative glomerulonephritis, and steroid therapy was initiated. After the worsening of the clinical picture, including an atypical skin rash, she was diagnosed with SARS-CoV-2. The re-evaluation of initial biopsy showed cytoplasmatic blebs and virus-like particles in tubular cells at electron microscopy. Despite SARS-CoV-2 clearance and the intensification of immunosuppression, no improvement was observed. A second kidney biopsy showed a crescentic glomerulonephritis with sclerosis, while virus-like particles were no longer evident.

The second patient was a 12-year-old girl with a 3-week history of weakness and weight loss. Rhinitis was reported the month before. No medications were being taken. Blood and urine analysis revealed elevated serum creatinine, hypouricemia, low molecular weight proteinuria, and glycosuria. A high SARS-CoV-2-IgG titre was detected. Kidney biopsy showed acute tubular-interstitial nephritis. Steroid therapy was started with a complete resolution of kidney involvement.

**Conclusion:**

We can speculate that in both cases SARS-CoV-2 played a major role as inflammatory trigger of the kidney damage. Therefore, we suggest investigating the potential kidney damage by SARS-CoV-2 in children. Moreover, SARS-CoV-2 can be included among infectious agents responsible for pediatric acute tubular interstitial nephritis.

## Introduction

The novel coronavirus (SARS-CoV-2) infection has rapidly become a pandemic, with an aggressive and even fatal course in adults with comorbidities, and milder clinical manifestations in children [1]. Children with chronic kidney disease (CKD) or on immunosuppression for nephrotic syndrome, glomerular diseases, or transplantation show neither a more severe clinical course nor an increased risk of infection, compared to healthy peers [2–5]. SARS-CoV-2 nephropathy has been reported in both healthy adults and children. Most common features include acute kidney injury, tubular-interstitial damage, proteinuria, and/or hematuria [6]. The exact mechanism of kidney involvement is unclear, and probably multifactorial. SARS-CoV-2 could directly damage tubular epithelial cells and podocytes as a result of a specific kidney tropism, through the Angiotensin-converting enzyme (ACE) receptors, present in high concentrations in the kidney, or indirectly trigger a cytokine storm associated with multi-organ failure and thrombotic events [6, 7]. ACE polymorphisms and the high-risk apolipoprotein L1 (APOL1) genotype variants have been recently described as genetic modifiers associated with a higher proinflammatory state and podocyte damage in patients with coronavirus diseases (COVID-19) [6]. Kidney biopsies from adults usually reveal acute tubular necrosis or thrombotic microangiopathy, collapsing glomerulopathy, and acute endothelial injury [8, 9], while data are lacking in children. Here we report two pediatric cases of SARS-CoV-2-related kidney involvement, documented by kidney biopsies.

## Case 1

**Fig. 1:**
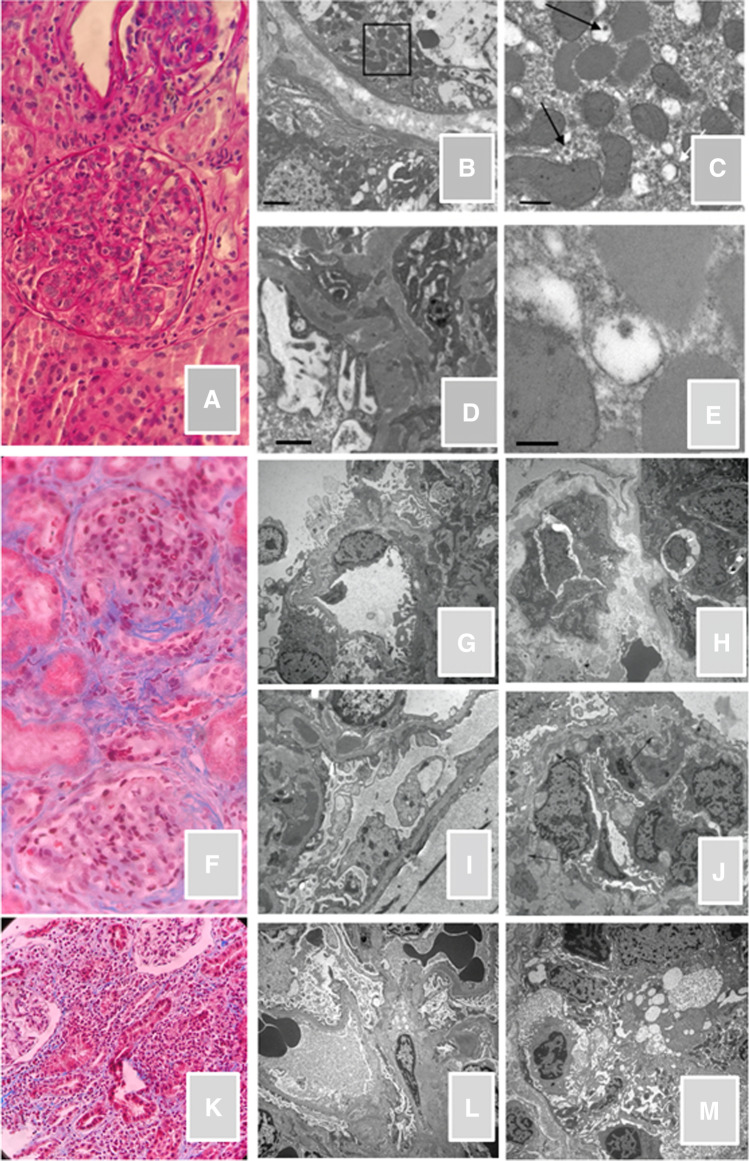
**A**, **B**, **C**, **D**, **E** Histological finding of first biopsy in patient 1. **A** (*light microscopy*): diffuse and segmental mesangial proliferation with endocapillary proliferation without crescents or sclerosis (periodic acid Schiff stain). *Electron microscopy*: a tubular cell (**B**) contains rare intra-vacuolar virus-like particles (detail **C**, arrows) of about 80–90 nm in diameter, with rare preserved spikes (**E**), structure, and location suggestive of coronavirus infection. The cell also contains isolated vesicles delimited by a double membrane, similar to the viral replicative organelle DMV involved in viral-RNA replication, described in RNA-positive virus included SARS-CoV2. OM (original magnification): **B** × 4400, **C** × 20,000, **D** × 85,000. Bar: **B** 2micron, **C** 500 nm, **D** 200 nm. **C** Glomerular fine granular electron dense deposits, with mesangial and sub-endothelial localization. **F**, **G**, **H**, **I**, **J** Histological finding of second kidney biopsy in patient 1. *Light microscopy*: evidence of global mesangial proliferation with fibrocellular crescent in 30% of glomeruli (of the 33 glomeruli, fibrocellular crescents were present in 9, and fibrotic crescents in 6, of which 2 have complete floccular sclerosis), diffuse segmental glomerular sclerosis, and initial membranoproliferative pattern in association with interstitial fibrosis, suggestive of a worsening of kidney activity and an appearance of signs of chronic nephropathy. Trichrome stain (**F**). *Electron microscopy*: increase in the sclerotic component of matrix (**G**), widespread presence of finely granular electron dense deposits (**H**, **I**, **J**); presence of segmental GBM deposits mainly intramembrane, sometimes sub-endothelial and occasionally sub-epithelial (**I**). Lamellation and reticulation of the lamina densa (**I**). Features of reabsorption of immune deposits (**H**, **I**). Focal images of deposits associated with mesangial interpositions (membranoproliferative pattern) (**G**, **J**). Podocytes: alterations secondary to lesions of the basement membrane. Segmental monocytes and rare granulocytes sometimes with occlusion of the capillary lumen, sometimes associated with intraluminal fibrin aggregates (**I**, **J**). Widespread loss of fenestrations of the endothelial cells (**I**). No virus-like particles were evident. **K**, **L**, **M** Histological features of kidney biopsy in patient 2. *Light microscopy*: marked neutrophilic, and lympho-plasmacellular invasion of the interstitium with multi-focal tubular acute damage suggestive of tubulitis. Trichrome stain (**K**). *Electron microscopy*: mild secondary sclerotic-ischemic changes of the mesangial matrix associated with mild thickening of GBM and podocytes effacement (**L**, **M**). No images suggestive of immune deposits or virus-like particles were observed

In February 2020, Henoch–Schönlein purpura (HSP) was diagnosed in a previously healthy 10-year-old girl, based on the typical cutaneous manifestations. Four weeks later, gross hematuria and nephrotic range proteinuria (urinary protein-to-creatinine ratio [uPCR] up to 8.7 g/g) appeared and a first kidney biopsy was performed (Fig. 1A–E). A diffuse and segmental mesangial-proliferative glomerulonephritis was detected. Immunofluorescence (IF) showed only a granular pattern of mesangial deposition of IgA (2 +), without signs of complement in situ activation. Two courses of intravenous methylprednisolone (15 mg/kg/day for 3 consecutive days) and oral prednisone (1 mg/kg on alternate days) were administered. Nevertheless, new evidence of skin vasculitic lesions, gross hematuria, mild kidney impairment (serum creatinine 0.78 mg/dl, eGFR 75 ml/min/1.73sqm) with hypoalbuminemia (serum albumin 2.29 g/dl), and proteinuria (uPCR 9.38 g/g) occurred. No concomitant infectious diseases were reported. Seven weeks after biopsy, the clinical conditions worsened with severe weight gain (up to 4 kg), persistence of nephritic-nephrotic syndrome, and the appearance of a distinct skin rash characterized by purpuric lesions and erythema pernio-like, mainly localized on feet and lower limbs. Because of the atypical presentation of the skin rash, and the growing awareness of skin involvement by COVID-19, a molecular nasopharyngeal swab test was performed, which tested positive for SARS-CoV-2. At admission, the patient was afebrile without clinical or radiological respiratory involvement. SARS-CoV-2 was undetectable on urinary samples. Parents were positive for SARS-CoV-2-IgG, with a negative swab test. Because of persistent nephrotic range proteinuria after clearance of SARS-CoV-2 infection, a third 3-daily pulse of methylprednisolone and oral cyclophosphamide (2 mg/kg/day for 10 weeks) were added to oral prednisone therapy.

The re-evaluation of electron microscopy (EM) on the kidney biopsy showed cytoplasmatic blebs and virus-like particles in tubular cells (Fig. 1C–E). Nonetheless, the RT-PCR for SARS-CoV-2 from the kidney tissue result was negative.

For the persistence of severe proteinuria (uPCR 2.81–3.4 g/g), 2 months later, a second kidney biopsy was performed, showing worsened active lesions with the appearance of crescents in almost 30% of the glomeruli (Fig. 1F–J), and chronic features including fibrocellular crescents, interstitial fibrosis, and diffuse segmental glomerular sclerosis. On EM, virus-like particles were no longer evident. RT-PCR for SARS-CoV-2 tested negative. Treatment with mycophenolate mofetil and ACE-inhibitors was started. At the last follow-up, 1 year after HSP-onset, the patient has normal kidney function (serum creatinine 0.65 mg/dl, eGFR 99 ml/min/1.73sqm) and low proteinuria (uPCR 0.36 g/g).

## Case 2

A previously healthy 12-year-old girl was referred to our department in June 2020, because of a 3-week history of progressive lack of appetite, weakness and weight loss (5 kg), and evidence of moderate kidney impairment (serum creatinine 1.2 mg/dl, eGFR 59 ml/min/1.73sqm). A mild rhinitis, without use of medications, was reported the month before. Blood and urine analysis confirmed an elevated serum creatinine of 1.8 mg/dl associated with hypouricemia (1.8 mg/dl), severe tubular proteinuria (uPCR 0.5 mg/mg, urinary beta-2-microglobulin 19,793 ug/L), low urinary gravity (1008), glycosuria, and ketonuria. Before hospital admission, the girl and her mother underwent a routine nasopharyngeal swab test for SARS-CoV2: the mother tested positive while the patient twice tested negative. Nonetheless, the patient showed a high SARS-CoV-2-IgG titre (195 AU/ml, cut-off > 11.9 AU/ml). Adenovirus, mycoplasma spp, streptococcus, cytomegalovirus, and Epstein-Barr virus were negative. SARS-CoV-2 was undetectable on urine. Acute tubulointerstitial nephritis (aTIN) related to SARS-CoV-2 infection was clinically suspected and confirmed by a kidney biopsy, showing marked neutrophilic, and lympho-plasmacellular invasion of the interstitium with multi-focal tubular acute damage suggestive of tubulitis (Fig. 1K–M). IF was negative. On EM, no images suggestive of immune deposits or for virus-like particles were observed. SARS-CoV-2 RT-PCR on kidney tissue was negative. Oral prednisone (1 mg/kg/day) was started, and 10 days after the admission, the girl was discharged. During the following 2 weeks, kidney dysfunction and tubular abnormalities resolved. Three months after the onset, therapy was stopped, and protective SARS-CoV-2-IgG titre persisted (21.1 cut off > 0.99 AU/ml).

## Discussion

SARS-COV-2 infection usually runs asymptomatic both in previously healthy and nephropatic children [1, 3–5]. Published data highlight both direct and indirect virus-related kidney damage [7]. Nevertheless, kidney involvement is uncommon in pediatric patients and the related histological data are lacking. AKI is the most feared kidney complication in children with COVID-19, but there is no consensus regarding its best management. To date, kidney biopsies are reported only in two children with COVID-19-associated AKI [10], showing an acute necrotizing glomerulonephritis in both cases. EM was not available. Similar to our patients, RT-PCR for SARS-CoV-2 tested negative in kidney specimens, as well as in urine. We describe for the first time the comprehensive kidney histological framework from two young girls with SARS-CoV-2 infection. In both cases, the lack of typical respiratory involvement resulted in a delayed diagnosis. Regardless, we presume that the inflammatory reaction exclusively involved the kidney in both girls.

In case 1, failure to gain remission of the underlying kidney disease and the new appearance of an atypical cutaneous rash suggested a SARS-CoV-2 infection, confirmed by the nasopharyngeal swab test. Indeed, pernio-like, macular erythema and vasculitis skin lesions are described in COVID-19 children, usually considered a late manifestation, lasting about 14 days, and associated with a more severe clinical course [11]. Therefore, cutaneous involvement of patient 1 suggested an infection occurred at least 2–3 weeks earlier that could overlap with the time of the first biopsy. Indeed, the presence of virus-like particles at the EM evaluation of first biopsy may be related to the presence of SARS-CoV-2 infection before the modification of cutaneous lesions and the worsening of kidney function. The latency between the onset of SARS-CoV-2-related symptoms despite an early evidence of viral elements at the kidney biopsy could be associated with the impaired immunological response secondary to persistent nephrotic proteinuria and to immunosuppressive therapy.

We can speculate that, despite the ongoing therapy, the pre-existing IgA vasculitis might have worsened due to the inflammatory trigger played by SARS-CoV-2. The histological findings mimicking viral particles in the first kidney biopsy and the evidence of a global worsening of the histological features of the second one suggest an association between SARS-CoV-2 infection and the severity of kidney inflammation. An acute exacerbation of a pre-existing kidney disease following COVID-19 was hypothesized also in one of the 2 cases described by Basiratnia et al. [10]. The better prognosis of our case may be related to the absence of general and respiratory involvement.

While different reports of COVID-19 in adults highlight the presence of SARS-CoV-2 on EM with distinctive spikes or virus-like particles in the tubular epithelium or podocytes [6–9, 12], this is the first description of similar features in children. However, viral inclusions are also reported as incidental findings, not necessarily implicated in kidney damage, and can be mimicked by many structures [6, 9, 13]. Despite indirect signs, SARS-CoV-2 RNA by RT-PCR in kidney tissue and urine were undetected in our patient. This is in line with the only other pediatric report by Basiratnia et al. [10]. However, the presence of the virus at a concentration level below the threshold of detection cannot be excluded.

SARS-CoV2-related tubular damage has already been described in COVID-19 adults [8]. In case 2, we describe for the first time the clinical and histological features of SARS-CoV-2-related aTIN, which appeared around 4–6 weeks after the first symptoms. In fact, SARS-CoV-2-IgG were already present at the time of kidney biopsy. aTIN is rare in children, usually caused by drug toxicity or viral infections [14, 15]. Similar to other infectious agents, the pathogenesis of tubular injury is unclear and may be associated with immunological changes established after direct or indirect damage by SARS-CoV-2. In our case, the latency between mild respiratory manifestations and the development of aTIN may suggest an indirect damage mediated by inflammatory cells, as also highlighted by the lympho-plasmacellular invasion at the kidney biopsy. As reported with other virus-triggered aTIN, viral particles are cleared before the clinical onset of kidney disease, characterized by a sterile tubular-interstitial infiltrate [14]. In fact, we were not able to demonstrate viral-like particles on EM. Therefore, SARS-CoV-2 can be added to the list of etiological infectious agents associated with aTIN [14, 15]. In our case, the response to steroids was excellent, with complete kidney recovery in 2 weeks. Considering the recent approval by the European Medicines Agency of a SARS-CoV-2 mRNA vaccine in children older than 12 years, we do not foresee contraindications to the vaccination in this girl, in accordance with the standard schedule for patients with a previous infection.

In conclusion, this is the first complete description of kidney histological findings and relative clinical pictures in two children infected by SARS-CoV-2 with only extrapulmonary manifestation. We strongly suggest further investigation into the potential kidney damage done by SARS-CoV-2 in children with or without clear pulmonary disease when other alarm signs appear, because kidney involvement can be sneaky and latent.
